# Cost-Effectiveness of Lenvatinib Plus Pembrolizumab or Everolimus as First-Line Treatment of Advanced Renal Cell Carcinoma

**DOI:** 10.3389/fonc.2022.853901

**Published:** 2022-06-21

**Authors:** Ye Wang, Hao Wang, Manman Yi, Zhou Han, Li Li

**Affiliations:** ^1^ Nanjing Drum Tower Hospital, China Pharmaceutical University, Nanjing, China; ^2^ Department of Pharmacy, Drum Tower Hospital Affiliated to Medical School of Nanjing University, Nanjing, China

**Keywords:** cost-effectiveness, lenvatinib, pembrolizumab, partitioned survival, renal cell carcinoma

## Abstract

**Background:**

In this study, compared to sunitinib as one of the available treatment options, we aimed to evaluate the cost-effectiveness of lenvatinib plus pembrolizumab or everolimus as first-line treatment for advanced renal cell carcinoma (RCC) patients in a Chinese health system setting.

**Methods:**

A partitioned survival model was developed to simulate patient disease and death. Transition probabilities and adverse reaction data were obtained from the CLEAR trial. The utility value was derived from literature. Costs were based on the Chinese drug database and local charges. Sensitivity analyses and were performed to assess the robustness of the model. Outcomes were measured as quality-adjusted life-years (QALYs), cumulative cost (COST), and incremental cost-effectiveness ratio (ICER).

**Results:**

The model predicted that the expected mean result in the lenvatinib plus pembrolizumab group (2.60 QALYs) was superior to that in the sunitinib group (2.13 QALYs) to obtain 0.47 QALYs, but the corresponding cost was 1,253,130 yuan greater, resulting in an ICER of 2,657,025 RMB/QALYs. Compared with the sunitinib group, the lenvatinib plus everolimus group (2.17 QALYs) gained 0.04 QALYs, with an additional cost of 32,851 yuan, resulting in an ICER of 77,6202 RMB/QALYs.

**Conclusions:**

Lenvatinib plus pembrolizumab or everolimus has no economic advantage over sunitinib in treating advanced RCC in the Chinese healthcare system.

## Introduction

As one of the most common cancers, the global incidence and mortality rate of renal cell carcinoma (RCC) has increased worldwide. The impact of renal cancer on human health in western countries and China should not be underestimated (1, 2). According to the Chinese Cancer Statistics, renal cancer has a high incidence and mortality of approximately 66,800 and 23,400, respectively, per year in China (3). Although the incidence of renal cancer is low in China, the incidence of renal cancer ranks first in the world. The Global Burden of Disease (GBD2019) data reveal that the number of disability-adjusted life years (DALYs) caused by renal cancer in China reached as high as 643,000, accounting for 0.17% of the total number of DALYs (4). Metastatic RCC accounts for more than 90% of renal cancers, is usually asymptomatic at the initial stage, has a poor prognosis, and has a five-year survival rate of only 11% (5). Furthermore, the medical expenditure and socioeconomic burden caused by renal cancer increases each year (6).

Sunitinib, a small molecule, multi-targeted tyrosine kinase inhibitor, is currently an effective tool for the treatment of advanced RCC as a first-line clinical treatment (7). In the NCCN Clinical Practice Guideline for Kidney Cancer, the panel includes first-line sunitinib as a category 2A, other recommended regimen for patients with ccRCC across all risk groups (8). According to ESMO (European Society for Medical Oncology) Clinical Practice Guideline, sunitinib is a potential alternative to PD-1 inhibitor-based combination therapy in IMDC favourable risk disease due to the lack of clear superiority of PD-1-based combinations over sunitinib in this subgroup of patients (9). Despite the initial response, most patients are prone to relapse as resistance develops. However, immune checkpoint inhibitors as dual therapy in combination with kinase inhibitors provide better outcomes than sunitinib (10–12). The recently published phase III clinical trial of CLEAR demonstrated the clinical benefit of lenvatinib plus pembrolizumab in the treatment of advanced RCC (13). The median progression-free survival (PFS) in the lenvatinib-plus-pembrolizumab vs. sunitinib was 23.9 vs. 9.2 months [hazard ratio (HR), 0.39; 95% confidence interval (CI), 0.32–0.49], and was longer with lenvatinib plus everolimus than with sunitinib (median, 14.7 vs. 9.2 months; HR, 0.65; 95% CI, 0.53–0.80). Overall survival (OS) was better with lenvatinib plus pembrolizumab (HR, 0.66; 95% CI, 0.49–0.88), and no longer with lenvatinib plus everolimus than with sunitinib (HR, 1.15; 95% CI, 0.88–1.50).

The trial results showed a superior survival advantage of lenvatinib in combination with pembrolizumab in advanced RCC, and this treatment was approved by the FDA (13). Furthermore, the Chinese Society of Clinical Oncology (CSCO) guidelines for renal cancer in 2021 have further recommended a combination regimen of lenvatinib plus pembrolizumab for grade IA RCC. Dual combination of immune checkpoint inhibitors and kinase inhibitors improves the health outcomes in patients with advanced RCC. However, questions concerning the associated substantial drug costs, adverse events, health benefits, and reduced consumption of health resources for subsequent therapies remain unresolved. The high cost of immune checkpoint inhibitors limits their use, especially in areas where health resources are scarce, and the cost-effectiveness of these new therapies requires further evaluation (14). After retrieval, there has been no economic study on the combination of lenvatinib plus pembrolizumab in China. The purpose of this study was thus to evaluate the cost-effectiveness of lenvatinib combined with pembrolizumab or everolimus and sunitinib as a first-line treatment for advanced renal carcinoma in Chinese patients. In the context of the healthcare system in China, this study provides evidence to support for patients, physicians, and policymakers in the treatment of advanced first-line renal cell cancer.

## Methods

### Model Overview

**Figure 1:**
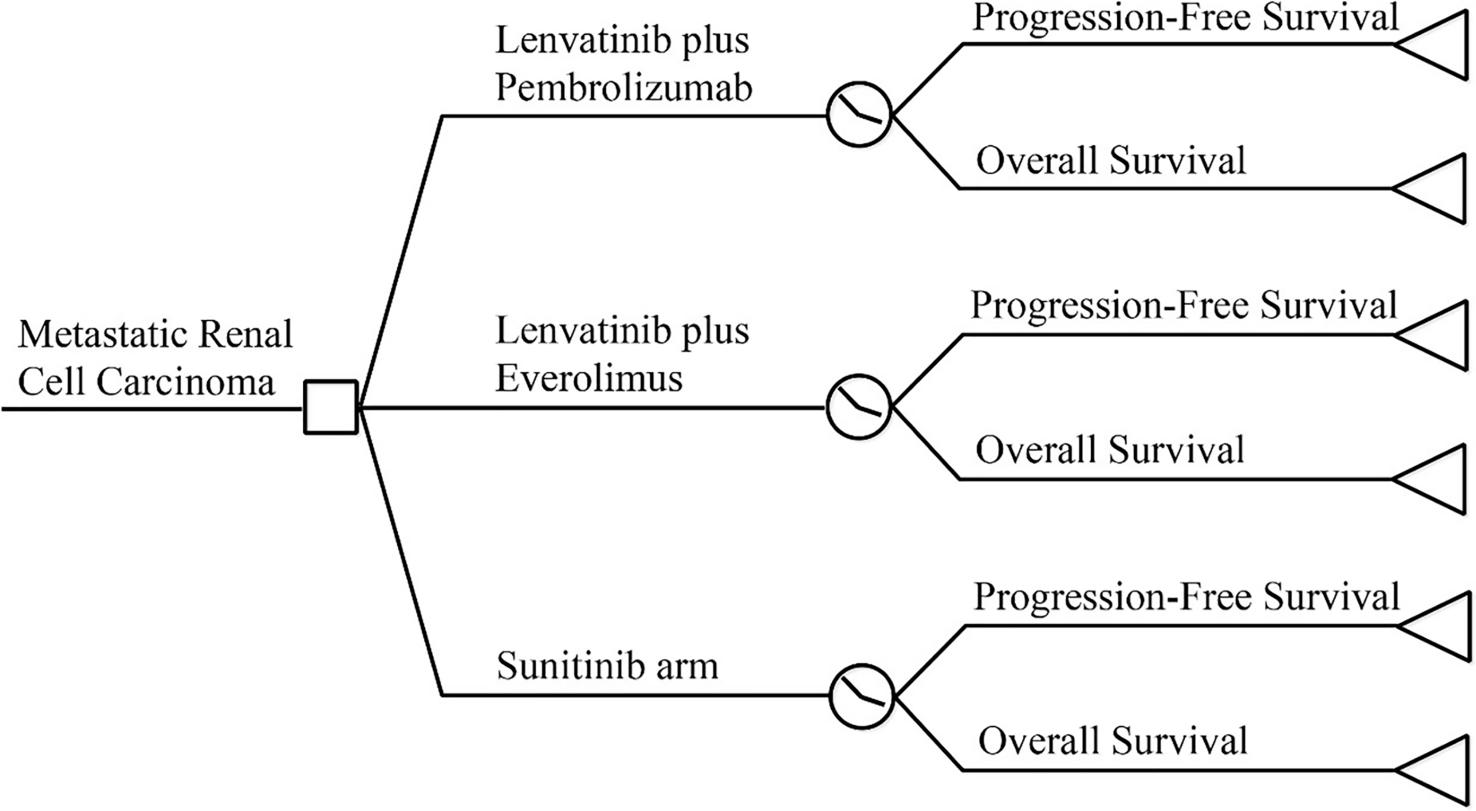
Model Structure.

A partitioned survival model was constructed using TreeageProSuit2020 to assess the economic benefits of lenvatinib in combination with pembrolizumab using a cost-utility approach from the perspective of the Chinese Health System (**Figure 1**). The model period was five years, and the study model only calculated direct medical costs, including drug costs, adverse event management costs, subsequent treatment costs after disease progression, follow-up costs, and hospital service program costs. The incidence of adverse events was estimated from the CLEAR (13) randomized controlled trial study, and the utility values were extracted from previous studies (14–16). The cost unit is expressed in local currency. An annual discount rate of 5% was applied to long-term costs and health utilities (17).

The model results were expressed as quality-adjusted life-years (QALYs), life-years (Lys), cumulative cost (COST), and incremental cost-effectiveness ratio (ICER). According to the China Guidelines for Pharmacoeconomic Evaluation issued by the Chinese Pharmaceutical Association (18), and one to three times the GDP per capita (RMB 217,341) in 2020 was used as the cost-effectiveness threshold. In addition, deterministic and probabilistic sensitivity analyses were performed to assess the model stability. As the economic analysis was based on literature review and experimental models, the approval of an institutional review board or ethics committee was not required.

### Partitioned Survival Model

The model was constructed using a partitioned survival model, a method currently used for health technology assessment to simulate disease status in patients with advanced RCC (19–21). The PS model is similar to the Markov model in that both characterize health states, and state membership is determined by a series of non-independent survival curves (22, 23). The three-state PS model is often used in the economic evaluation of advanced solid tumors, including progression-free disease, progressive disease, and death absorption states. All the patients were characterized as being in a progression-free state until disease progression and death at the start of the simulation. The patient received subsequent therapy after disease progression until death. The reconstructed survival curve was used to estimate the proportion of members in each state. The upper part of OS represents the dead patient, the lower part of PFS represents the disease-free patients, and the part between PFS and OS represents the progressed and surviving patients (21).

### Clinical Data

The inclusion criteria and treatment regimen for the study target population were consistent with the CLEAR trial (13). We assumed that all patients were progression-free and received one of the following treatments at the beginning of the model: lenvatinib 20 mg orally once daily and pembrolizumab 100 mg intravenously on each 21-day treatment cycle; everolimus 5 mg and lenvatinib 18 mg orally once daily; sunitinib 50 mg orally once daily for 28 days followed by 14 days without treatment.

### Curve Fit

Kaplan–Meier (KM) survival curves for the three treatment regimens were extracted from the CLEAR trial using Engauge Digitizer version 12.2.2. According to Guyot et al. (24), individual data were reconstructed by combining KM curve information with the number of people at risk of events using the survHE package in the R language (4.0.4). Exponential, Weibull, Gompertz, Gen-Gamma, log-logistic and log-normal distribution functions were used to extrapolate the probability of survival to cover the lifetime horizon. First, PFS and OS were visually examined and compared to those in the original report. The optimal fitting distribution was judged according to the Bayesian information criterion (BIC) and Akaike information criterion (AIC) (25). In comparison, the study ultimately fitted individual patient data using Weibull distribution and log-logistic analysis (see **Table S1** and **Figure S1**).

### Medical Costs

Direct medical costs were calculated in a Chinese health system setting, this comprised: drug treatment regimen costs, adverse event management costs, follow-up costs, hospital service item costs, and post-progression drug treatment costs, as shown in **Table 1**. The drug cost is mainly calculated according to the bid price and the usage and dosage in the package insert. The cost of drugs that have been marketed we include in health insurance according to the price of drugs, and the cost of drugs comes from China Pharmaceutical Information Network-Menet (www.menet.com). For the cost of drugs not yet marketed in China, we use a public database.

**Table 1:** Summary of main medical costs, utility values, and other parameters.

Parameters	Base case	Range	Distribution	Source
**Drug cost (¥)**				
Pembrolizumab/mg	179.18	143.34-215.02	Gamma	MENET
Lenvatinib/table	108	86.4-129.6	Gamma	MENET
Everolimus/table	130	104-156	Gamma	MENET
Sunitinib	98	78.4-117.6	Gamma	MENET
LenvPemb subsequent treatment cost/per cycle	6416	5132-7699	Gamma	MENET
LenvEver subsequent treatment cost/per cycle	16301	19561-13040	Gamma	MENET
Sunitinib subsequent treatment cost/per cycle	17461	13969-20953	Gamma	MENET
**Subsequent treatment proportion**				
Lenvatinib+Pembrolizumab	0.549	0.44-0.66	Beta	(13)
Lenvatinib+Everolimus	0.68	0.55-0.82	Beta	(13)
Sunitinib	0.71	0.57-0.85	Beta	(13)
**Probability of treatment discontinuation as a result of AE (%)**				
Lenvatinib+Pembrolizumab	0.372	0.298-0.446	Beta	(13)
Lenvatinib+Everolimus	0.27	0.216-0.324	Beta	(13)
Sunitinib	0.144	0.115-0.173	Beta	(13)
**Follow-up cost/cycle (¥)**				
Complete blood count	18	14-21	Gamma	Local charge
CT	220	176-264	Gamma	Local charge
Bioch	214	171-257	Gamma	Local charge
Urine	10	8-12	Gamma	Local charge
Consultation fee	12	9.6-14.4	Gamma	Local charge
**Management cost/cycle (¥)**				
Bed	50	40-60	Gamma	Local charge
Care	27	21.6-32.4	Gamma	Local charge
Hoex	15	12-18	Gamma	Local charge
Trans	10	8-12	Gamma	Local charge
Preparation	40	32-48	Gamma	Local charge
Best support care	353	282-423	Gamma	(26)
Terminal care	12721	10177-152665	Gamma	(26)
**Management of Aes (¥)**				
Diarrhea	276	220-331	Gamma	(26)
Hypertension	80.4	64-96	Gamma	(26)
Decrease dappetite	705.4	564-846	Gamma	(26)
Nausea	298	238-357	Gamma	(26)
Vomiting	298	238-357	Gamma	(26)
Proteinuria	775	620-930	Gamma	(26)
Palmarplantar	102	82-122	Gamma	(26)
Rash	294	235-352	Gamma	(26)
**Utility**				
Progression-free survival	0.82	0.656-0.984	Beta	(14–16)
Sunitinib Progression-free survival	0.73	0.584-0.876	Beta	(14–16)
Progressive disease	0.66	0.528-0.792	Beta	(14–16)
Disutility due to AEs (grade ≥3)	0.157	0.126-0.188	Beta	(14–16)
**Others**				
Discount rate	5%	0–8%	Fixed in PSA	(18)

CT, computed tomography; Hoex, hospitalization examination.

According to the recommendations of the NICE economic technology (27), subsequent treatment and its proportion were derived from the supplementary data of the CLEAR trial. After discontinuation of first-line therapy, 54.9% of the patients in the lenvatinib plus pembrolizumab arm received subsequent therapy, with 68.2% and 71% doing the same in the lenvatinib plus everolimus and the sunitinib arm, respectively. The most common subsequent therapies used in the experiments were antiangiogenic agents and PD-1 or PD-L1 inhibitors. The model cost calculation did not include the cost of drugs that have not yet been marketed in China. For the availability and convenience of the model, the following drugs were considered: nivolumab 240 mg intravenously on each two weeks treatment cycle and pazopanib 800 mg orally once daily.

Accompanying adverse events should not be ignored during the use of immune checkpoint inhibitors and antiangiogenic drugs. Here, only the management cost of grade III and above adverse events was considered for the cost of adverse events, including diarrhea, hypertension, decreased appetite, nausea, vomiting, proteinuria, erythema syndrome, and rash. The incidence of adverse drug reactions was derived from phase III clinical trials. The processing costs of adverse drug reactions were derived from recent publications (26, 28). We also captured the cost of AEs by administering a questionnaire to clinical experts.

### Utility Values

Health utility scores were collected from the public literature (14, 16, 26). The PFS status utility was 0.82 for the lenvatinib plus pembrolizumab or everolimus groups and 0.73 for the sunitinib group, while the PD status utility was 0.66. Weights of disutility values arising from adverse events with three or more levels were included in the model. QALYs were estimated using weighted patient survival based on utility calculations for each health state. The main medical costs, utility values, and other model parameters are listed in **Table 1**.

### Sensitivity Analyses

One-way sensitivity analysis and probabilistic sensitivity analysis (PSA) were performed to assess the model’s stability and analyze the effect of each parameter change on the model. The upper and lower limits of the cost parameters were derived from the China Pharmaceutical Information Database and Medical Service Price Standards. The minimum and maximum values of each parameter are listed in **Table 1**. A reasonable range of discount rates was set as 0%–8% (18). The results of the univariate sensitivity analysis were expressed as tornado plots. In the probability sensitivity analysis, Monte Carlo simulation conducted 10,000 iterations of cost and utility parameters. The cost and utility value parameters were set to gamma and beta distribution, respectively. Scatter plots and cost-effectiveness acceptance curves are used to present the results of different competitive strategies, showing the possibility of cost-effectiveness at various levels of willingness to pay (WTA) threshold.

Scenario and subgroup analysis were performed to simulate the cost-effectiveness of competitive strategies in natural environments. Firstly, we changed the time range of 5, 10, and 20 to evaluate the impact of the extrapolation of the survival curve in the PS model. In the second scenario, we varied the price of first-line pembrolizumab to assess its impact on the ICER. In the absence of survival curves for each subgroup, it was assumed that all patients in the trial had the same baseline as the survival curve in the sunitinib group. The subgroup-specific HR was applied to estimate the survival curve for the lenvatinib plus pembrolizumab group according to Hoyle et al. (29).

## Results

### Base Case Analysis

**Figure 2:**
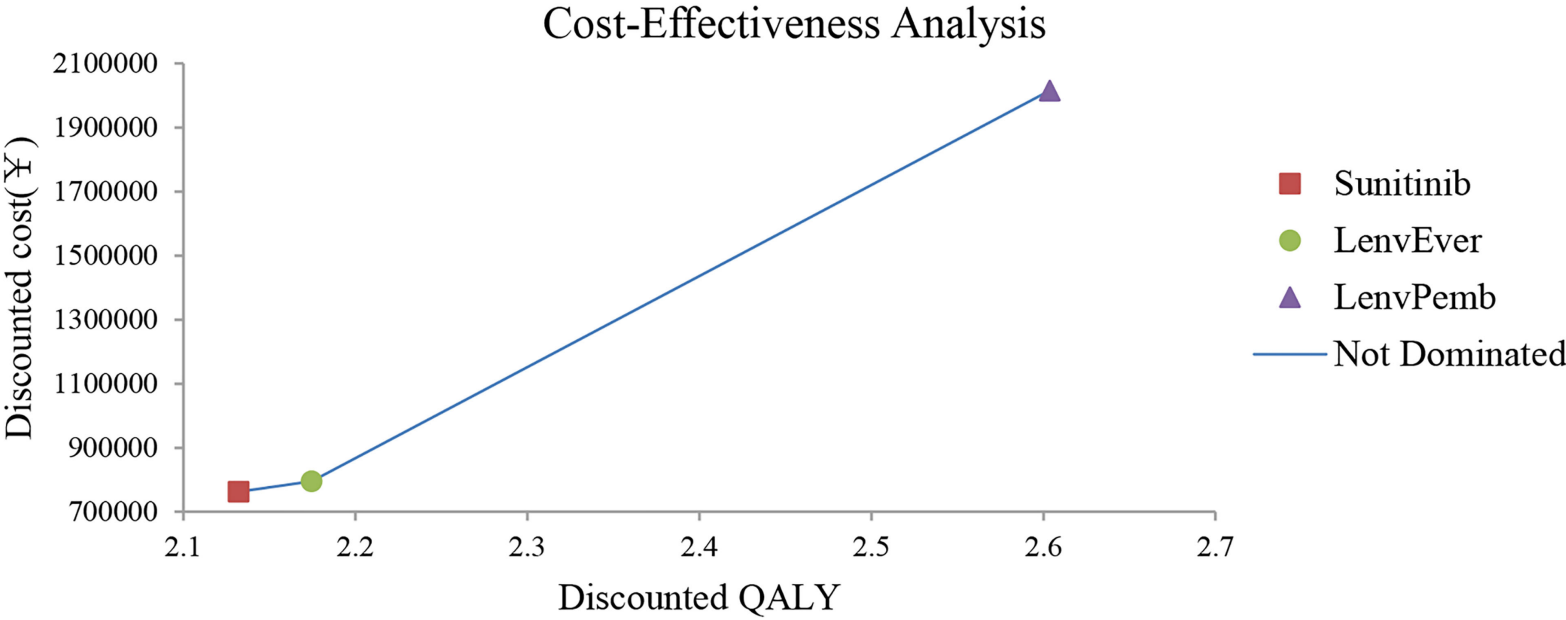
Results of the base-case analysis in the Partitioned Survival model. LenvPemb, Lenvatinib-plus-Pembrolizumab; LenvEver, Lenvatinib-plus-Everolimus.

The model predicted that the expected mean result in the lenvatinib plus pembrolizumab group (2.60 QALYs) was superior to that in the sunitinib group (2.13 QALYs) to obtain 0.47 QALYs, but the corresponding cost was 1,253,130 yuan greater, resulting in an ICER of 2,657,025 RMB/QALYs. Compared with the sunitinib group, the lenvatinib plus everolimus group (2.17 QALYs) gained 0.04 QALYs, with an additional cost of 32,851 yuan, resulting in an ICER of 77,6202 RMB/QALYs. The results of the fundamental analysis are presented in **Table 2** and **Figure 2**.

**Table 2:** Results of the base-case analysis.

	LY	QALY	Incremental QALY	Cost (RMB)	Incremental cost	ICER (RMB/QALY)
Sunitinib	2.83	2.13	–	762,572	–	–
Lenvatinib+Everolimus	2.96	2.17	0.04	795,424	32,851	776,202
Lenvatinib+Pembrolizumab	3.44	2.60	0.47	2,015,702	1,253,130	2,657,025

LY, life year; QALY, quality-adjusted life-year; ICER, incremental cost-effectiveness ratio.

### One-Way Sensitivity and Probability Analyses

**Figure 3:**
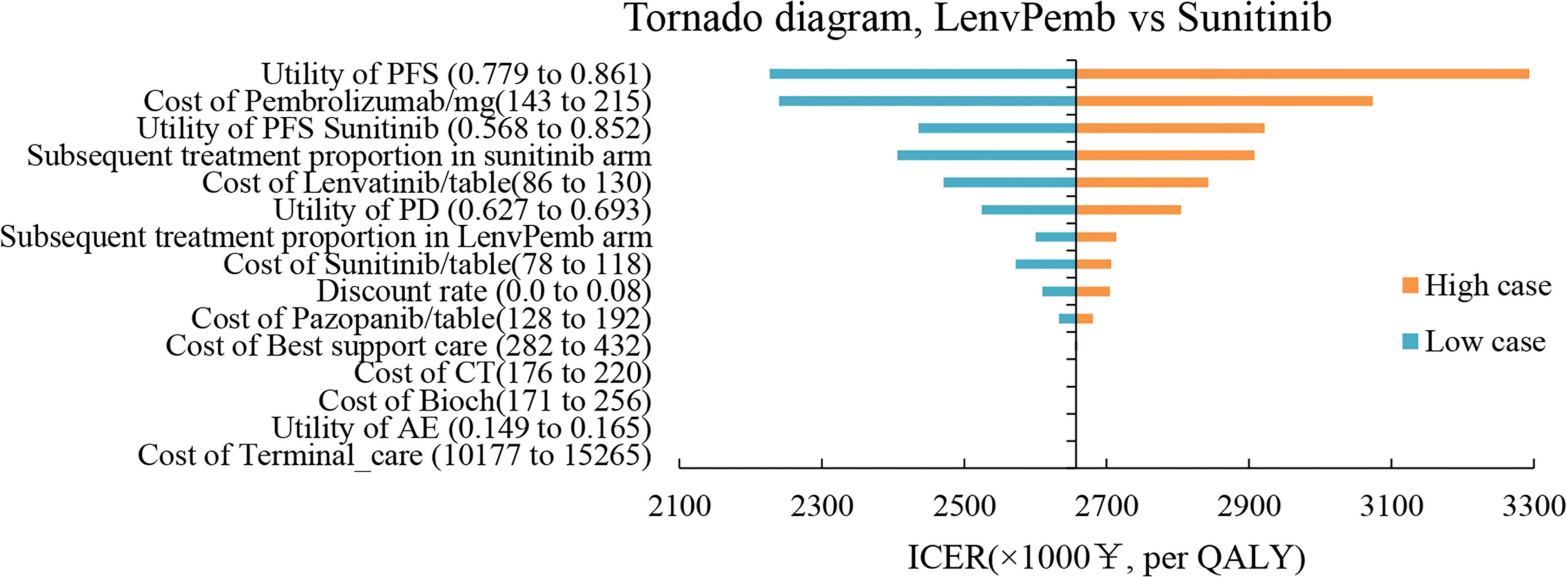
Tornado Diagrams Showing the Effect of Lower and Upper Values of Each Parameter on the ICERs of the Lenvatinib-plus-Pembrolizumab Versus Sunitinib Strategy.

**Figure 4:**
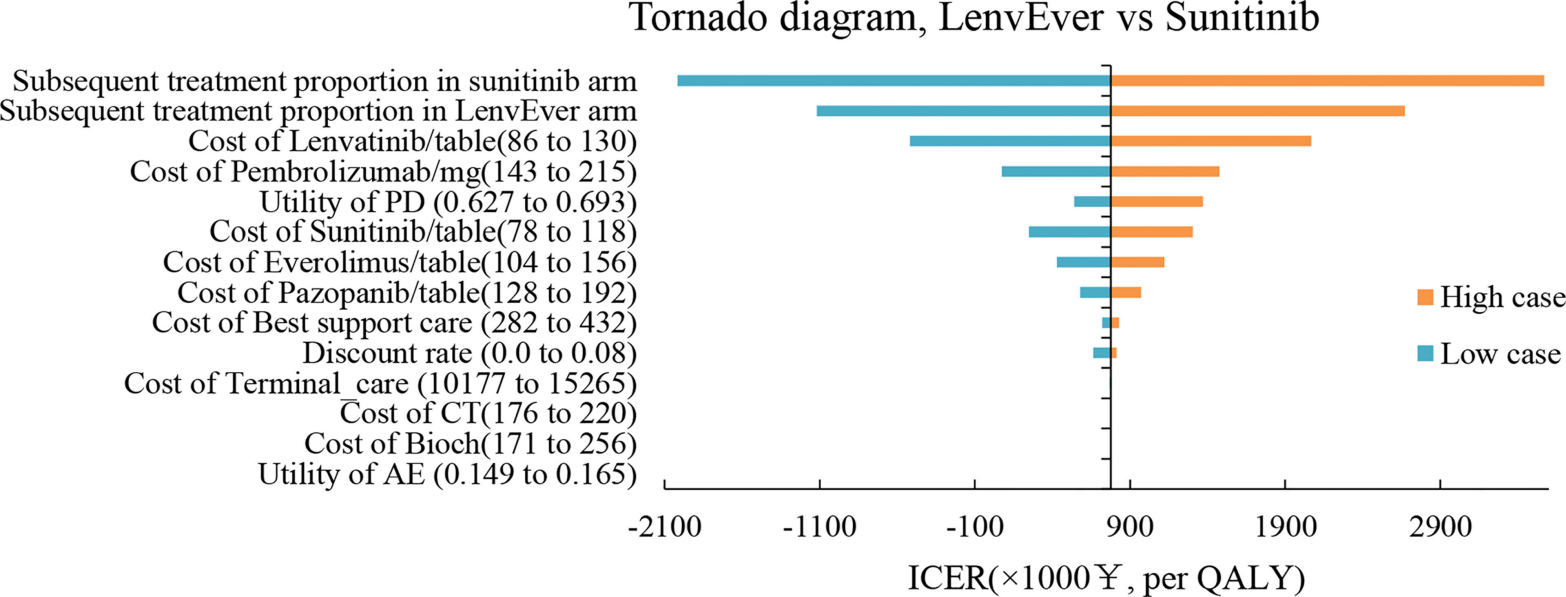
Tornado Diagrams Showing the Effect of Lower and Upper Values of Each Parameter on the ICERs of the Lenvatinib-plus-Everolimus Versus Sunitinib Strategy.

The results of the one-way sensitivity analysis are shown in **Figures 3**, **4** respectively. When comparing the lenvatinib plus pembrolizumab and sunitinib strategies, the most significant effect on the entire model was the utility value at the PFS stage, followed by the price of pembrolizumab. The ICER value changed from 2,226,657 to 3,293,613 RMB/QALY in the sensitivity analysis. The proportion of subsequent treatments with a more significant impact on the ICER was higher in the lenvatinib plus everolimus group than in the sunitinib group. Other PS model parameters had moderate or negligible effects on the expected ICERs.

**Figure 5:**
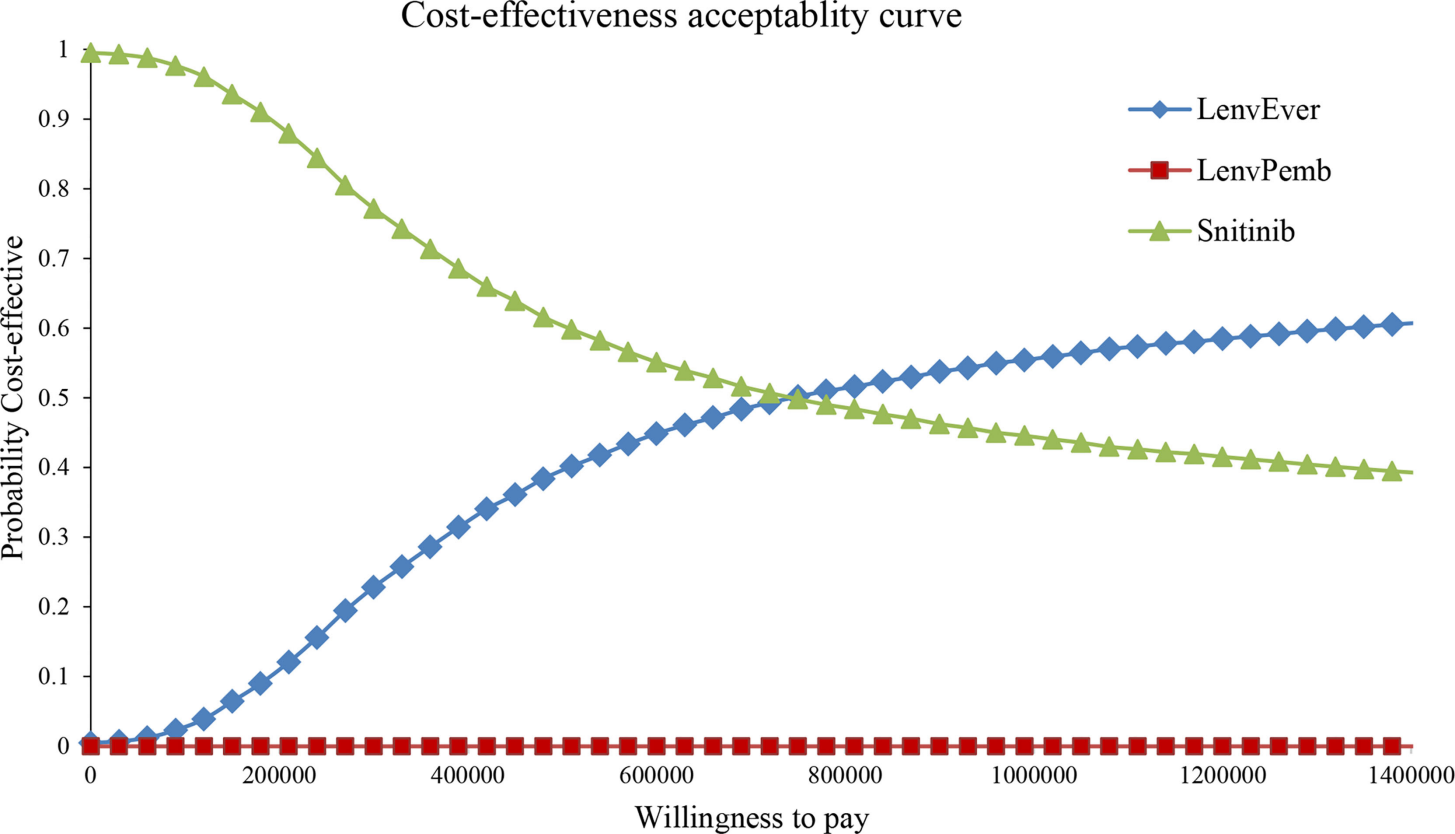
Cost-effectiveness acceptability curve in the Partitioned Survival model. LenvPemb, Lenvatinib-plus-Pembrolizumab; LenvEver, Lenvatinib-plus-Everolimus.

In the PSA analysis, all scatter distributions were above the willingness-to-pay threshold for lenvatinib plus pembrolizumab vs sunitinib. Lenvatinib plus everolimus was 57.6% more likely to be above the first quadrant and 14.3% more likely to be cost-effective below the WTA threshold than sunitinib strategies; see **Figures S2**, **S3** for details. The cost-effectiveness acceptability curve showed that the lenvatinib plus everolimus strategy was a cost-effective option with a 50% probability compared with sunitinib at a WTP threshold of 750,000 RMB/QALY (see **Figure 5**). Lenvatinib plus pembrolizumab was not cost-effective.

### Scenario and Subgroup Analyses

Scenario analysis reveals the variation of calculated ICER values in different natural environments (**Table S2**). When the model extrapolated years changed to 5, 10, and 20 years, an interesting phenomenon occurred, with 80% of the medical costs of patients spent in the first 5 years, after which there was still a clinical benefit. In the final scenario analysis, it was found that the purchase price of pembrolizumab used in first-line therapy was reduced by 25%, 50%, and 75%, resulting in a reduction in ICER of 673210.31, 1407276.31, and 2141343.31 RMB/QALY, respectively.

The QALY and cumulative cost of subgroup analysis were closely related to the heterogeneity of HR. All calculated ICER values remained above 1,500,000 RMB/QALY above the WTP threshold. In the group with a PD-L1-positive score≥1, the estimated ICER was 1,904,086.03 RMB/QALY, and in the group with a poor risk of International Metastatic RCC Database Consortium (IMDC), an additional 0.87 QALY was added, and the ICER was 1,514,440.51 RMB/QALY (see **Table S3**).

## Discussion

The advent of immune checkpoint inhibitors has led to new options for metastatic RCC (10–12, 30). Tyrosinase inhibitors combined with immune checkpoint inhibitors appear promising for RCC metastasis relative to the conventional standard of care treatment with sunitinib, with the potential for long-term durable benefits in patients. This regimen was also supported by the latest Chinese Society of Clinical Oncology (CSCO) in 2021, which recommended a combination regimen of lenvatinib plus pembrolizumab for grade IA RCC, regardless of IMDC risk. This study showed that, among the three competitive strategies, the lenvatinib plus pembrolizumab regimen significantly improved health outcomes in the early stages. However, the price of pembrolizumab in China remains remarkably high, and the disadvantage caused by such a massive cost gap cannot be compensated for by its clinical production. Thus, from a purely economic perspective, sunitinib is a superior treatment option for patients with metastatic RCC.

The findings are consistent with those of other cost-effectiveness analyses of pembrolizumab-containing regimens for advanced RCC. The strategy of pembrolizumab was not cost-effective in the treatment of advanced RCC, emphasizing substantial price reductions to rationally allocate health care resources. In a Chinese study report, Chen et al. (26) constructed a Markov model. They found that the ICER of pembrolizumab plus axitinib compared to sunitinib in advanced RCC was $55,185/QALY, which was much higher than the 1–3 times GDP threshold. Li et al. (16) constructed a network meta-analysis (NMA) and microscopic decision tree model for cost-effective analysis of the advanced first-line renal cell cancer. The health benefit of lenvatinib plus pembrolizumab was 2.61 QALY, and ICER did not dominate compared with sunitinib. However, there are several differences between this study and ours. The NMA is the preferred evidence synthesis method to evaluate multiple interventions across trials (31, 32). However, the application of NMA alleviates but does not eliminate the confusion caused by the cross-trial heterogeneity of the trial protocol and patient baseline (33). The choice of the microscopic decision tree model and subsequent treatment is also a fundamental reason for the differences in QALYs calculated by the studies.

The model was constructed using a PS structure with certain advantages, which is a well-established modeling approach to simulate metastatic RCC disease progression and death (19–21). Compared to the Markov model, PS model does not need to make assumptions about the probability of metastasis; only two outcomes, progression-free survival and overall survival, are required to inform on health status occupancy, and the time to progression status was inferred by the difference between the two outcomes (34). PS model can more accurately model disease events, avoid natural mortality, and access individual patient data. The primary disadvantage is that it can only be used in the process by which a patient progresses through a series of progressive health states. PS model, which is also widely understood by clinicians and other stakeholders, is the most common structure for applying the Health Technology Assessment in cancer treatment.

In the base analysis, the model period was five years. Five-year survival is the response assessment criterion that best reflects the value of immune combination therapy, and most healthcare costs (80%) are spent in the five years of the period. In the first scenario analysis, we further adjusted the model period to reflect the situation in long-term clinical practice; lenvatinib plus everolimus had some health benefits at five years, but no clinical benefit over sunitinib after ten years. The main reason for the change in QALY differences between the two strategies was that lenvatinib plus everolimus OS curves were inferior to those of sunitinib. However, lenvatinib + pembrolizumab still met the need for long-term survival benefits, and the ICER was consistently reduced with prolonged tracking time.

In the sensitivity analysis, the essential input parameter driving the model was pembrolizumab cost. In addition to drug costs, the proportion of drug treatment after disease progression and utility value of PFS also had a moderate impact on the model results. According to our model, the most realistic means of reducing the cost of the lenvatinib plus pembrolizumab strategy proportional to its clinical value is to reduce the price of pembrolizumab and lenvatinib. We further reflected the clinical reality through a scenario analysis. Substantial price reductions (25–75%) of pembrolizumab used in first-line therapy can result in ICERs well below baseline outcomes. Currently, both lenvatinib and pembrolizumab are commercially marketed in China. Lenvatinib was included in the Chinese Medical Insurance Catalog to achieve a price reduction of 80.7% in 2020. Assuming that pembrolizumab is successfully included in a new round of health insurance negotiations, Chinese health insurance companies will reimburse all cancer treatment costs. In this case, the lenvatinib plus pembrolizumab regimen will be an excellent treatment option for patients.

In the subgroup analysis, the influencing model variables were PFS HR and OS HR, which indicates that the cost-effectiveness of the lenvatinib plus pembrolizumab regimen could be improved by identifying specific patient populations. Patients with unfavorable IMDC risk or high tumor burden seemed to be candidates who could obtain better cost-effectiveness of the lenvatinib plus pembrolizumab regimen, with an ICER reduction of 43% in the group with poor-risk IMDC. PD-L1 expression is also an essential factor affecting ICER; a lower ICER may be obtained using the lenvatinib plus pembrolizumab regimen in patients with high PD-L1 expression. Nevertheless, the ICER value after the reduction is still much higher than the threshold of three times the GDP per capita in China.

This study has several limitations. First, our extrapolation of clinical data from the CLEAR trial inevitably brings a range of uncertainties, and any bias within the trial will be reflected in the current study. A series of sensitivity and scenario analyses were performed to assess uncertainty, but the true efficacy of lenvatinib combined with pembrolizumab remains an open question (35). It is necessary to evaluate the consistency of these modeled health outcomes with the long-term efficacy of real-world data. Second, subsequent treatment costs after disease progression were estimated based on information published from the CLEAR trial, which may differ from clinical practice in China and may or may not reflect the real-world prevalence of second-line therapy. Third, we used the quality of life (QOL) score of mRCC in western populations, which cannot truly reflect the data of Chinese patients. The study showed no significant difference in QOL between Asian and European populations. The robustness and accuracy of the model will be improved in future health utility analyses of patients with advanced RCC.

## Conclusions

According to the basic and sensitivity analysis results, lenvatinib combined with pembrolizumab or everolimus has no economic advantage over sunitinib in treating advanced RCC in the Chinese health care system. The combination of lenvatinib and pembrolizumab may benefit patients with advanced kidney cancer, but incurs additional costs. Our findings may support efforts to reduce drug prices and enable this treatment to reduce the economic burden on the Chinese health care system.

## Data Availability Statement

The original contributions presented in the study are included in the article/**Supplementary Material**. Further inquiries can be directed to the corresponding author.

## Author Contributions

Contributions: (I) Conception and design: YW and HW; (II) Administrative support: LL; (III) Provision of study materials or patients: YW and ZH; (IV) Collection and assembly of data: MY and YW; (V) Data analysis and interpretation: YW, HW, and MY; (VI) Manuscript writing: All authors; (VII) Final approval of the manuscript: All authors.

## Conflict of Interest

The authors declare that the research was conducted in the absence of any commercial or financial relationships that could be construed as a potential conflict of interest.

## Publisher’s Note

All claims expressed in this article are solely those of the authors and do not necessarily represent those of their affiliated organizations, or those of the publisher, the editors and the reviewers. Any product that may be evaluated in this article, or claim that may be made by its manufacturer, is not guaranteed or endorsed by the publisher.
