# Supplementary material for: Cost-Effectiveness of Lenvatinib Plus Pembrolizumab or Everolimus as First-Line Treatment of Advanced Renal Cell Carcinoma
Source: Front Oncol. 2022 Jun 21;12:853901. doi: 10.3389/fonc.2022.853901 (PMC9254865; doi:10.3389/fonc.2022.853901)
Supplement: Supplementary file 1 [file DataSheet_1.docx]

Supplementary Material

Cost-effectiveness of lenvatinib plus pembrolizumab or everolimus as first-line treatment of advanced renal cell carcinoma

**Figure S1.** Results of the survival curve fit the LenvPemb arm, LenvEver arm and Sunitinib arm

**Figure S2.** Scatter Plot showing the incremental cost-effectiveness ratio of the Lenvatinib-plus-Pembrolizumab Versus Sunitinib Strategy

**Figure S3.** Scatter Plot showing the incremental cost-effectiveness ratio of the Lenvatinib-plus-Everolimus Versus Sunitinib Strategy.

**Table S1** Best fit and the values of the parameters

**Table S2** Results of scenario analyses.

**Table S3** Results for subgroup analyses (Lenvatinib+Pembrolizumab)

**Firgure S1**


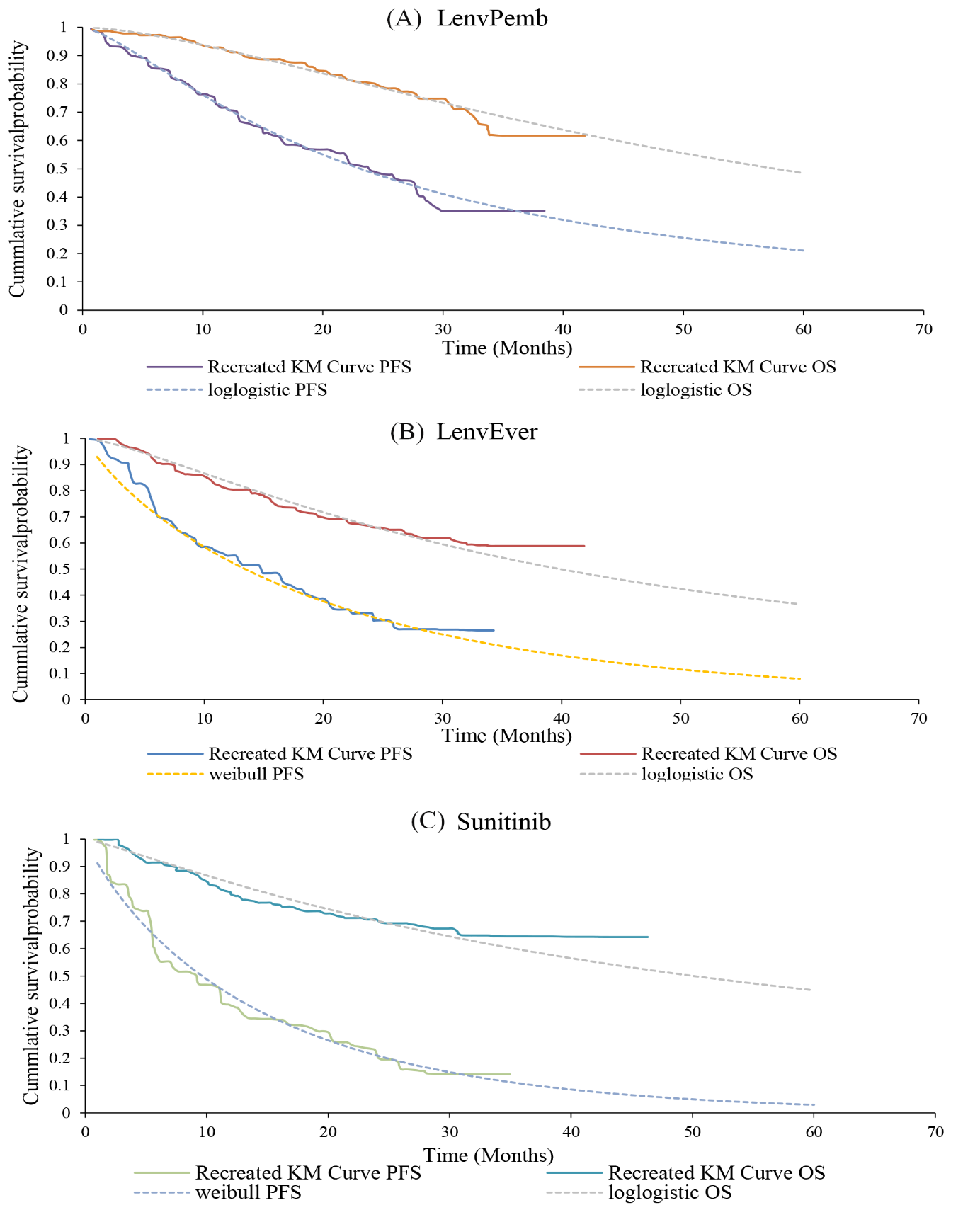


**Figure S1.** Results of the survival curve fit the LenvPemb arm, LenvEver arm and Sunitinib arm.

**Firgure S2**


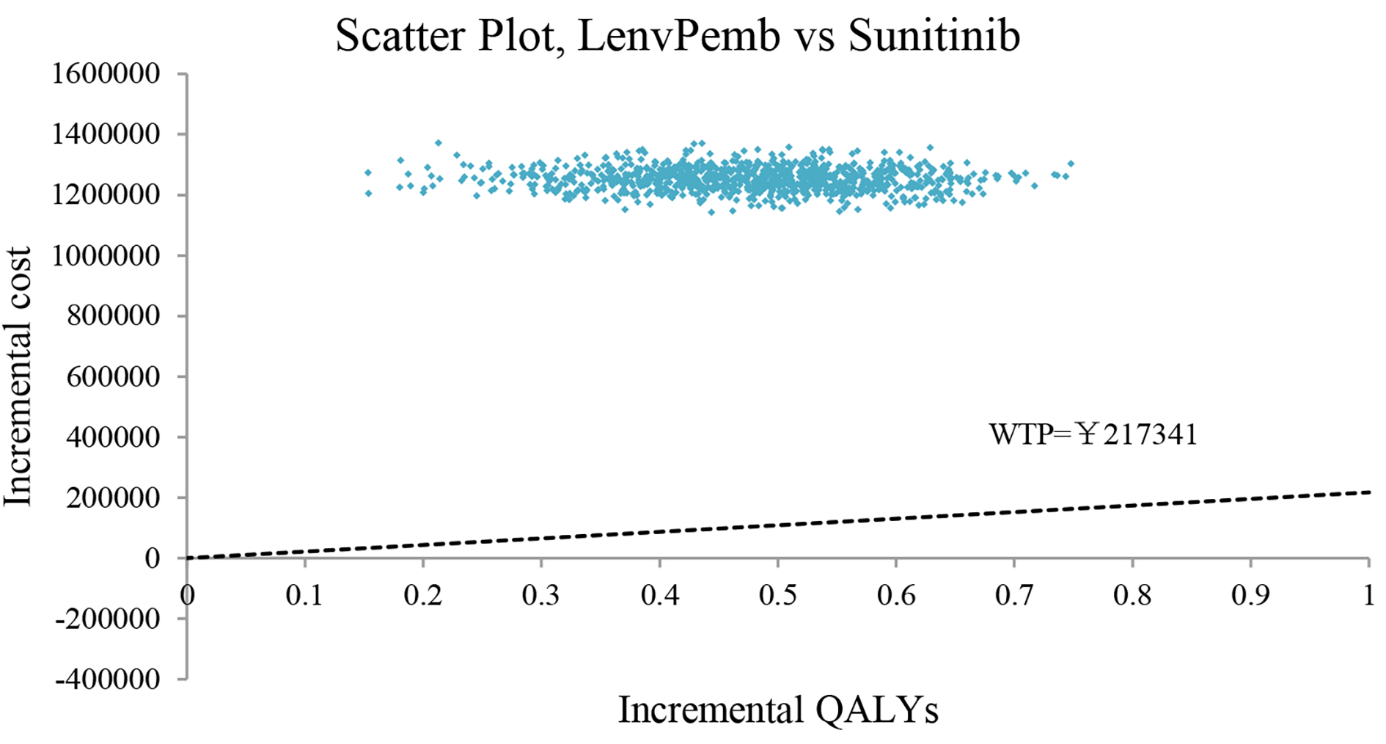


**Figure S2.** Scatter Plot showing the incremental cost-effectiveness ratio of the Lenvatinib-plus-Pembrolizumab Versus Sunitinib Strategy.

**Firgure S3**


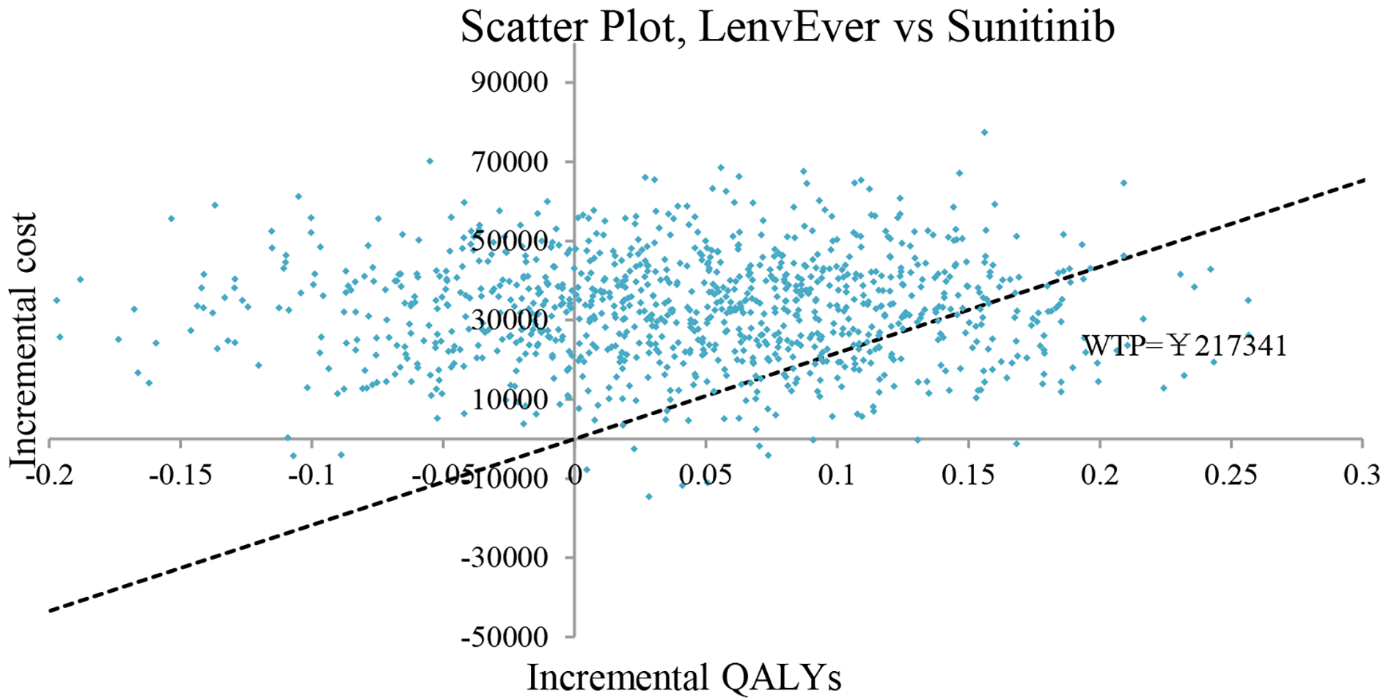


**Figure S3.** Scatter Plot showing the incremental cost-effectiveness ratio of the Lenvatinib-plus-Everolimus Versus Sunitinib Strategy.

**Table S1.** Best fit and the values of the parameters

|  | Best fitting | Parameters | Parameters | AIC | BIC |
| --- | --- | --- | --- | --- | --- |
| LEN+PEM PFS | Log-logistic | shape=1.38403 | scale=23.12299 | 1423.348 | 1431.092 |
| LEN+PEM OS | Log-logistic | shape=1.54418 | scale=57.65644 | 883.4196 | 891.1638 |
| LEN+EVE PFS | Log-logistic | shape=1.48123 | scale=13.76222 | 1505.344 | 1513.1 |
| LEN+EVE OS | Weibull | shape=1.18928 | scale=52.47358 | 1216.663 | 1224.419 |
| Sunitinib PFS | Weibull | shape=1.12548 | scale=14.52788 | 1476.789 | 1484.545 |
| Sunitinib OS | Log-logistic | shape=1.16178 | scale=50.08289 | 1069.91 | 1077.665 |

LEN+PEM, Lenvatinib plus Pembrolizumab arm; LEN+EVE, Lenvatinib plus Everolimus; PFS, progression-free survival; OS, overall survival; AIC, Akaike information criterion; BIC, Bayesian information criterion.

**Table S2.** Results of scenario analyses.

| Strategy | Total cost | LY | QALY | ICER |
| --- | --- | --- | --- | --- |
| Scenario 1 | | | | |
| 10 years | | | | |
| Lenvatinib+Everolimus | 1054938 | 3.85 | 2.78 | - |
| Sunitinib | 1140106 | 4.33 | 2.92 | 631935 |
| Lenvatinib+Pembrolizumab | 2510228 | 4.63 | 3.47 | 2125883 |
| 15 years | | | | |
| Lenvatinib+Everolimus | 1171992 | 4.24 | 3.04 | - |
| Sunitinib | 1334075 | 4.92 | 3.31 | 605276.52 |
| Lenvatinib+Pembrolizumab | 2714358 | 5.15 | 3.83 | 1947616.24 |
| 20 years | | | | |
| Lenvatinib+Everolimus | 1233484 | 4.45 | 3.18 | - |
| Sunitinib | 1444720 | 5.27 | 3.54 | 592451.26 |
| Lenvatinib+Pembrolizumab | 2816839 | 5.40 | 4.02 | 1882315.23 |
| Scenario 2 | | | | |
| Adjust Pembrolizumab 75% of its original price in the first-line setting. | | | | |
| Sunitinib | 730213 | - | 2.13 | - |
| Lenvatinib+Pembrolizumab | 1665556 | - | 2.60 | 1983217.97 |
| Adjust Pembrolizumab 50% of its original price in the first-line setting. | | | | |
| Sunitinib | 730213 | - | 2.13 | - |
| Lenvatinib+Pembrolizumab | 1319349 | - | 2.60 | 1249151.07 |
| Adjust Pembrolizumab 25% of its original price in the first-line setting. | | | | |
| Sunitinib | 730213 | - | 2.13 | - |
| Lenvatinib+Pembrolizumab | 973141 | - | 2.60 | 515084.17 |

**Table S3.** Results for subgroup analyses (Lenvatinib+Pembrolizumab)

| Subgroup | OS HR (95% CI) | PFS HR (95% CI) | Incremental QALY | ICER (RMB/ QALY) |
| --- | --- | --- | --- | --- |
| Age |  |  |  |  |
| < 65 years | 0.63 (0.41-0.95) | 0.37 (0.28–0.49) | 0.53 | 2296232.76 |
| ≥ 65 years | 0.61 (0.40-0.95) | 0.43 (0.31–0.61) | 0.55 | 2152154.27 |
| Sex |  |  |  |  |
| Male | 0.70 (0.49-0.99) | 0.38 (0.30–0.49) | 0.50 | 2568734.44 |
| Female | 0.54 (0.30-0.94) | 0.42 (0.27–0.66) | 0.64 | 2047298.61 |
| Geographic region | |  |  |  |
| Western Europe and North America | 0.68 (0.46-1.00) | 0.42 (0.32–0.57) | 0.61 | 1968344.32 |
| Rest of the world | 0.63 (0.40-0.99) | 0.36 (0.26–0.49) | 0.53 | 2236220.28 |
| MSKCC risk group | |  |  |  |
| Favorable | 0.86 (0.38-1.92) | 0.36 (0.23–0.54) | 0.38 | 3384431.60 |
| Intermediate | 0.66 (0.47-0.94) | 0.44 (0.34–0.58) | 0.54 | 2460787.40 |
| Poor | 0.50 (0.23-1.08) | 0.18 (0.08–0.42) | 0.64 | 1828511.67 |
| IMDC risk category | |  |  |  |
| Favorable | 1.15 (0.55-2.40) | 0.41 (0.28–0.62) | 0.29 | 5794864.09 |
| Intermediate | 0.72 (0.50-1.05) | 0.39 (0.29–0.52) | 0.46 | 2589975.94 |
| Poor | 0.30 (0.14-0.64) | 0.28 (0.13–0.60) | 0.87 | 1514440.51 |
| Karnofsky performance-status score | |  |  |  |
| 90 or 100 | 0.73 (0.52-1.03) | 0.38 (0.30–0.48) | 0.54 | 2895047.94 |
| 70 or 80 | 0.48 (0.26-0.87) | 0.44 (0.26–0.74) | 0.69 | 1894690.91 |
| No. of organs with metastases | |  |  |  |
| 1 | 0.88 (0.47-1.67) | 0.46 (0.30–0.71) | 0.33 | 3396910.20 |
| ≥ 2 | 0.56 (0.40-0.79) | 0.36 (0.28–0.47) | 0.58 | 1947487.23 |
| PD-L1 combined positive score | |  |  |  |
| < 1 | 0.76 (0.46-1.27) | 0.40 (0.27–0.58) | 0.46 | 2861799.98 |
| ≥ 1 | 0.50 (0.28-0.89) | 0.39 (0.26–0.59) | 0.66 | 1904086.03 |

CI, confidence interval; HR, hazard ratio; IMDC, International Metastatic RCC Database Consortium;
